# Caregiver perceptions of telemedicine in developmental and epileptic encephalopathies: a nationwide Spanish survey

**DOI:** 10.3389/fneur.2026.1842836

**Published:** 2026-09-28

**Authors:** Simona Giorgi, Sandra Solaz, David Alarcón-Alarcón, Aroa Arboleya, Elena Cardenal-Muñoz, Isabel Manzanares, María Palanca Cámara, Irene Sánchez-Miranda, Antonio Gil-Nagel, José Ángel Aibar

**Affiliations:** 1 Dravet Syndrome Foundation Spain, Madrid, Spain; 2 Department of Health, Hospital La Fe, Silla Health Center, Silla, Valencia, Spain; 3 Department of Biomedical Sciences, Universidad Cardenal Herrera-CEU, CEU Universities, Elche, Spain; 4 Faculty of Nursing, University of Barcelona, L'Hospitalet de Llobregat, Barcelona, Spain; 5 Refractory Epilepsy Unit, Hospital La Fe, Silla, Valencia, Spain; 6 Hospital Ruber International, Madrid, Spain

**Keywords:** DEEs, healthcare resources, patient satisfaction, real world evidence, telemedicine

## Abstract

**Introduction:**

Developmental and epileptic encephalopathies (DEEs) require intensive and multidisciplinary care. Telemedicine has emerged as a potential tool to improve access and reduce burden for caregivers and healthcare professionals (HCPs), but its acceptance and perceived value in severe epilepsies among caregivers and HCPs remain unclear.

**Methods:**

Two online surveys (August–November 2024) targeted caregivers of Spanish patients with DEEs and HCPs treating patients with DEEs practicing in Spain. Due to low HCP response (*n* = 18, patients’ age: 0–54 years old), only caregiver data (*n* = 95) were analyzed.

**Results:**

Caregivers (98% parents, 78% female) reported high healthcare demands and a preference for increased medical contact. Half used telemedicine, mainly telephone (47%) or email (20%), but satisfaction was significantly lower than with in-person care (mean 3.2 vs. 4.6 on 5-point scale; *t*(94) = −7.96, *p* < 0.001). Logistical benefits (time saving, reduced travel) were highly valued (77% satisfied), whereas perceived clinical benefit for the patient was low. Rehabilitative therapies (speech, occupational) showed stronger preference for in-person format (>50%). In clinical trials (prior participation 41%), telemedicine was seen as improving comfort and accessibility but was limited by absence of physical examination (24%) and personal interaction.

**Discussion:**

Among caregivers of patients with DEEs, telemedicine was valued for its logistical benefits but not considered clinically equivalent, particularly for rehabilitative therapies and physical examination. These results should be interpreted cautiously, as telemedicine use in our sample relied predominantly on telephone and email, with very limited adoption of videoconferencing, the standard modality in neurological telemedicine, which restricts conclusions about its clinical equivalence. Tailored hybrid models preserving in-person high-complexity care and improved digital strategies are needed to address caregiver expectations and service-specific requirements. Findings should be considered exploratory and hypothesis-generating rather than confirmatory.

## 1 Introduction

Developmental and epileptic encephalopathies (DEEs), including Dravet syndrome (DS), are complex neurological disorders marked by early-onset, drug-resistant seizures, cognitive and motor impairments, and a range of comorbidities such as behavioral and language difficulties (1, 2). Although telemedicine gained traction during the COVID-19 pandemic to maintain continuity of care, its implementation and perceived value vary significantly across patient populations (3–5). Large epilepsy cohorts have shown heterogeneous acceptance of telemedicine, with videoconferencing yielding higher satisfaction than telephone-only models, and caregiver preferences influenced by child age, clinical complexity, and type of service (e.g., neurology vs. rehabilitation). Telemedicine tends to be better received in advisory or follow-up contexts but remains more controversial for developmental and rehabilitative therapies, where physical examination and patient-clinician interaction are critical.

In Spain, telemedicine for epilepsy during the COVID-19 lockdown was predominantly telephone-based and showed high acceptance among neurologists (6) yet only 58% of Dravet syndrome (DS) caregivers perceived remote assessments as valuable (7). Most post-pandemic evidence still comes from general epilepsy or adult populations; data specific to severe pediatric DEEs remain scarce.

Patients with DEEs often present with complex comorbidities that are difficult to assess through phone calls alone. Furthermore, the motor, cognitive, behavioral and rehabilitative needs of DEE patients are challenging to evaluate and treat remotely, raising doubts about the suitability of telemedicine beyond simple follow-up. As Bluvstein & Wenniger emphasized, optimal care for DS patients requires close collaboration between clinicians and caregivers, and a nuanced understanding of the syndrome’s progression across different life stages (8). Existing literature shows that videoconferencing is considered the standard telemedicine modality for neurological assessment, as it allows direct behavioral observation and real-time interaction, whereas telephone-only contact limits the clinical assessment (9, 10). Despite this, little is known about how caregivers of children with DEEs experience these different remote formats or which modalities are perceived as meaningful in high-complexity care.

We aimed to assess current telemedicine use, caregiver satisfaction, service-specific preferences, and attitudes toward its application in clinical trials among Spanish families of patients with DEEs and healthcare professionals (HCPs). In the present study, telemedicine was defined broadly to include telephone calls, e-mail, WhatsApp, healthcare mobile application and videoconferencing because these modalities represented the forms of remote care available in Spain. However, we acknowledge that videoconferencing is generally regarded as the reference modality for neurological telemedicine because it allows visual assessment and real-time interaction.

## 2 Methods

### 2.1 Study design

This is a cross-sectional, observational study based on two anonymous online surveys conducted in Spain from 1 August to 30 November 2024. Target populations were (i) primary caregivers of patients with developmental and epileptic encephalopathies (DEEs) residing in Spain and (ii) HCPs with clinical experience in the care of DEE patients in Spain. Although DEEs typically begin in childhood, affected individuals frequently require lifelong care; therefore, caregivers of both pediatric and adult patients were eligible.

This survey study is reported in accordance with the Consensus-based Checklist for Reporting of Survey Studies (CROSS) reporting guideline (11).

### 2.2 Participants and recruitment

A convenience sampling strategy was employed. Recruitment was carried out through the *Fundación Síndrome de Dravet* (FSD), in collaboration with other Spanish patient advocacy organizations (PAOs) such as *Asociación de afectados CDKL5*, *Asociación KCNQ2 España*, *Asociación GNAO1 España*, among others. PAGs invited participants via email and through their social media platforms (WhatsApp, Facebook, X, Instagram, and LinkedIn). For the survey directed to HCPs, dissemination was done through EpiCARE and the Spanish General Council of Nursing.

Both surveys were hosted on the FSD website. Geographic diversity across Spanish regions was actively encouraged during dissemination. Participants were recruited throughout Spain through national patient advocacy organizations, professional networks, and social media dissemination strategies, and no geographical restrictions were applied. Throughout this manuscript, the term “nationwide” refers to the national scope of recruitment and participation rather than statistical representativeness of the Spanish DEE population.

Although the initial design aimed to compare caregiver and HCP perspectives, the number of HCP responses (*n* = 18) did not provide sufficient statistical power for group comparisons. Including these data in inferential analyses risked producing unstable or misleading estimates. For transparency, healthcare professional responses are presented descriptively in the Supplementary material. Given the limited sample size (*n* = 18), they were not included in inferential analyses or caregiver-professional comparisons. All results presented hereafter are based exclusively on the caregiver cohort (*n* = 95).

### 2.3 Survey development

For the purposes of the survey, telemedicine encompassed any healthcare interaction conducted remotely, including telephone calls, e-mail communication and videoconferencing. The survey instrument underwent expert review and cognitive pretesting, but it did not undergo formal psychometric validation. The questionnaires were purpose-designed by the study investigators (neurologists, nurses, patient advocacy representatives, and a primary-care physician) using an iterative process. Items were derived from published literature on telemedicine in epilepsy and rare diseases, existing validated caregiver-burden instruments, and focus-group input from FSD staff and families. Draft versions underwent two rounds of review by the co-author group and cognitive pre-testing with two caregivers to ensure clarity and comprehension. Final versions were locked before data collection began. The cognitive pretesting involved two caregivers selected through the FSD, one with prior experience using telemedicine and one without, to ensure variability in digital literacy. They assessed item clarity, comprehensibility, and response burden, leading to minor refinement in wording and order of selected questions. The complete questionnaires (English translation) are provided as Supplementary material (Supplementary questionnaires. Survey for Professionals and Survey for Caregivers).

### 2.4 Variables

The survey collected variables across four main domains: (i) sociodemographic and clinical characteristics; (ii) healthcare utilization and caregiver expectations; (iii) telemedicine exposure and perceptions; (iv) attitudes toward telemedicine in clinical trial settings. Attitudinal items were measured using Likert-type scales (0–4 or 1–10 depending on the question), with satisfaction scores analyzed as continuous variables and categorical responses summarized as frequencies and percentages. Specialty-specific analyses were limited by sample size; therefore, visit types were grouped globally, with service-level differences explored descriptively when feasible.

### 2.5 Statistical analysis

Analyses were performed using R (version 4.3.2). Categorical data are reported as frequencies (%). Continuous data are presented as mean ± SD or median (IQR) as appropriate. Differences in satisfaction between telemedicine and in-person care were assessed using paired t-tests. Between-modality satisfaction was compared by one-way ANOVA with Tukey post-hoc tests. Associations between categorical variables were examined using *χ*^2^ or Fisher’s exact tests. Multiple testing was controlled using Benjamini–Hochberg FDR. Missing data (<3% for key variables) were handled by listwise deletion. Two-sided *p* < 0.05 was considered significant. The analysis plan was pre-specified. Because this was an exploratory survey study, no formal power analysis was performed. The findings should therefore be interpreted as descriptive and hypothesis-generating.

### 2.6 Procedures and ethical considerations

The study was conducted in accordance with the principles outlined in the Declaration of Helsinki and received approval from the Ethics Committee for Research with Medicinal Products of the Hospital Universitario y Politécnico La Fe in Valencia, Spain (Reg. No. 2022-235-1).

All participants received a detailed information sheet and provided electronic informed consent prior to accessing the surveys. In compliance with the European Union’s General Data Protection Regulation (GDPR), personal data were anonymized and dissociated from the study results to ensure participant confidentiality.

## 3 Results

### 3.1 Participant characteristics and healthcare utilization

Ninety-five caregivers completed the survey (response rate could not be calculated due to open dissemination). Most were parents/legal guardians (97.9%), predominantly female (77.9%), with a mean age of 47.1 ± 8.3 years (Figure 1A). Patients (49 males, 46 females) had a median age of 14 years (IQR 7–20, range 0–54) (Figure 1B), showing a bimodal pattern. A detailed summary of demographic and clinical characteristics of the patient sample is provided in Supplementary Table S1. Dravet syndrome accounted for 71.6% of diagnoses, followed by KCNQ2-related DEE (11.6%). Participants resided in 28 Spanish provinces, with highest representation from Madrid (20.0%) and Barcelona (10.5%) (Figure 1C). Responses were obtained from multiple autonomous communities across Spain, supporting the nationwide scope of survey dissemination and participation.

**Figure 1:**
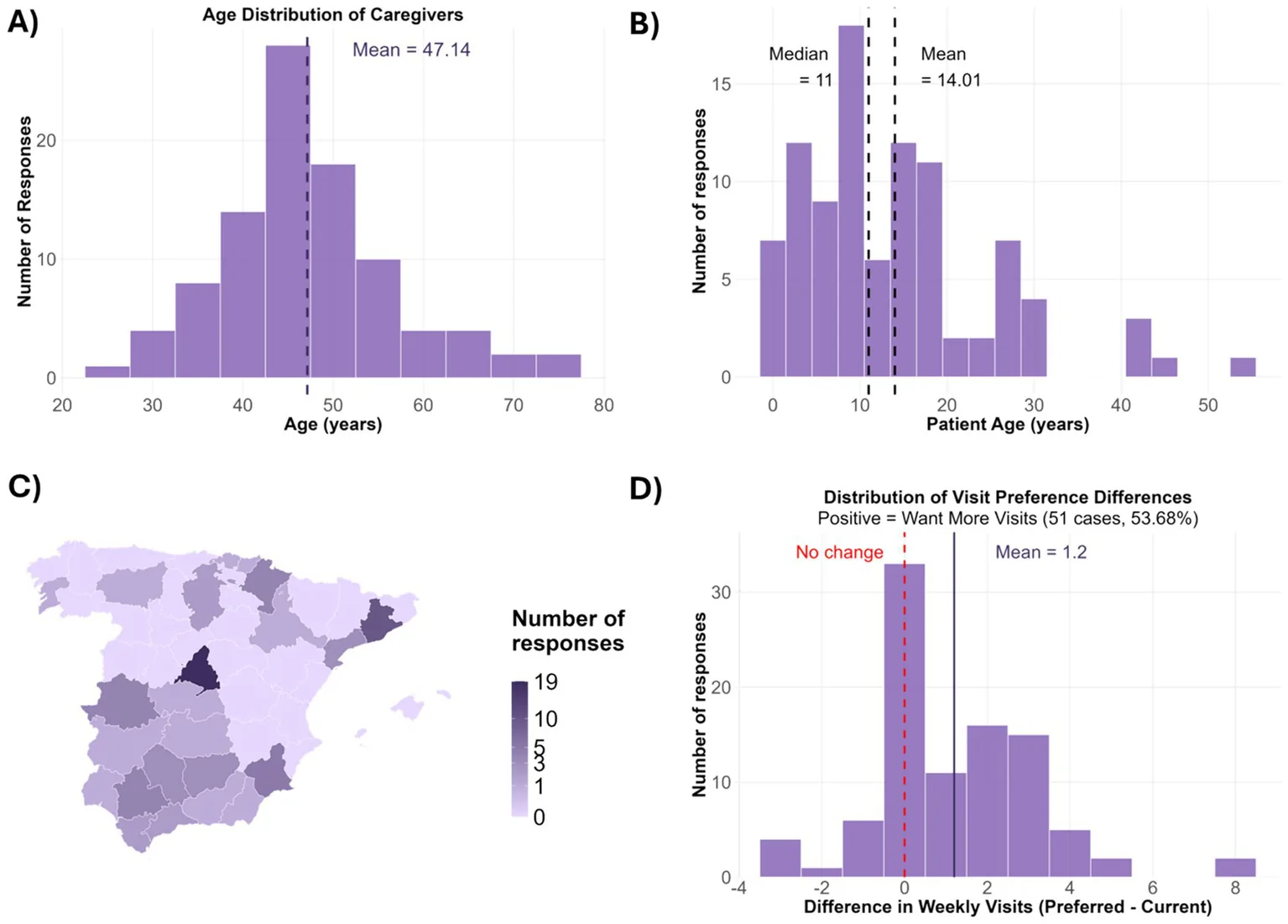
Sociodemographic and healthcare-related characteristics of caregivers and patients with DEEs (*n* = 95). **(A)** Age distribution of caregivers responding. **(B)** Age distribution of patients. **(C)** Geographical distribution of participants across Spanish provinces. **(D)** Comparison between current and preferred weekly medical visits, highlighting caregiver expectations.

Families reported a median of 2 (IQR 1–4) weekly healthcare visits of all types; the desired frequency was 4 (IQR 3–6) (*p* < 0.001, Wilcoxon signed-rank test). Only 3.2% of caregivers were satisfied with zero additional weekly visits (Figure 1D).

### 3.2 Descriptive findings from HCPs

Eighteen healthcare professionals completed the survey. Given the limited sample size, results were analyzed descriptively and are presented in Supplementary Figure S1.

Overall, HCPs reported favorable attitudes toward telemedicine. Most respondents agreed that telemedicine facilitates access to healthcare, were comfortable conducting remote consultations, and expressed openness toward future use of telemedicine. Telemedicine was also generally perceived as beneficial for patients with DEEs, although enthusiasm for replacing in-person care was limited. Among professionals performing remote visits, the most frequently reported challenges were the need for multiple call or connection attempts, difficulties related to patient or caregiver understanding of instructions, connectivity problems, and insufficient preparation of patients or families before the consultation. Very few respondents reported experiencing no difficulties during telemedicine encounters.

### 3.3 Telemedicine exposure and overall satisfaction

Forty-eight caregivers (50.5%) had used telemedicine. Among users, the most common modalities were telephone calls (47%) and e-mail (20%). Only 14 participants (16%) reported using videoconferencing, the modality considered standard in telemedicine, which substantially limits the ability to draw strong conclusions about the perceived value of full audiovisual remote care (Figure 2A).

**Figure 2:**
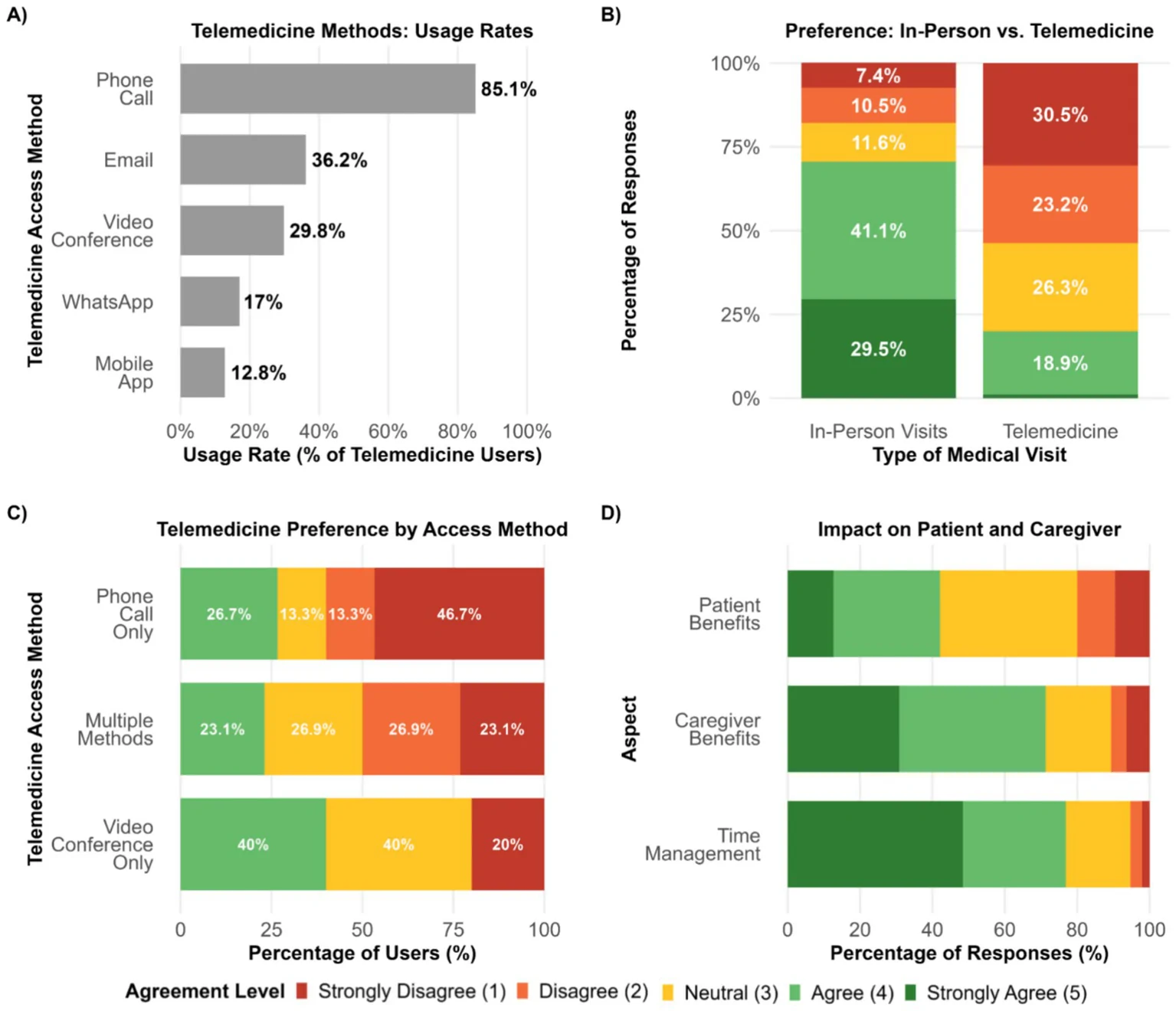
Use, satisfaction, and perceived impact of telemedicine among caregivers and patients with DEEs. **(A)** Telemedicine adoption and access modalities among telemedicine users (*n* = 47; multiple responses allowed). **(B)** Satisfaction with in-person visits and telemedicine visits measured on a 5-point Likert scale (1 = strongly disagree to 5 = strongly agree) (*n* = 95). Satisfaction was significantly higher for in-person visits than for telemedicine visits (paired t-test, *t*(94) = −7.96, *p* < 0.001). **(C)** Satisfaction according to telemedicine access modality among users with available satisfaction data (*n* = 46). The category “Multiple Methods” includes combinations of telephone calls, e-mail communication, videoconferencing, mobile applications, and/or messaging platforms. No significant differences in satisfaction were observed between modalities (one-way ANOVA, *F* = 0.887, *p* = 0.419). **(D)** Caregiver-reported perceptions of the impact of telemedicine on patient-related benefits, caregiver-related benefits, and time management. Responses are presented using the same 5-point Likert scale across panels B–D (1 = strongly disagree, 2 = disagree, 3 = neutral, 4 = agree, 5 = strongly agree). Percentages were calculated using valid responses for each item.

Overall satisfaction was significantly lower for telemedicine than for in-person care [mean 3.2 ± 1.1 vs. 4.6 ± 0.7 on a 5-point scale; paired t-test t (94) = −7.96, *p* < 0.001] (Figure 2B).

Satisfaction did not differ significantly across telemedicine modalities (one-way ANOVA *F* = 0.89, *p* = 0.42). Among telemedicine users, 40% of those who used videoconferencing rated it “satisfied/very satisfied,” compared with 26.7% of telephone-only users and 23.1% of mixed-modality users. However, differences were not statistically significant (one-way ANOVA *F* (2,82) = 0.89, *p* = 0.42) (Figure 2C). The “multiple methods” category comprised combinations of telephone calls, e-mail communication, videoconferencing, mobile applications and/or messaging platforms; videoconferencing was included within this category for participants who reported using more than one telemedicine modality.

### 3.4 Perceived benefits and limitations

Perceptions of telemedicine impact differed markedly between caregiver and patient. Items related to caregiver burden (time saving, reduced travel, logistical ease) received the highest ratings (>75% high/very high satisfaction), whereas items assessing direct clinical benefit to the patient (thorough evaluation, treatment efficacy) were rated substantially lower (<35% high/very high satisfaction) (Figure 2D).

When stratifying preferences according to healthcare service, notable differences emerged across disciplines (Supplementary Figure S2). Rehabilitative services such as speech therapy (52.05%) and occupational therapy (51.43%) showed the strongest preference for in-person care, reflecting the importance of direct interaction and hands-on assessment. In contrast, neurology consultations demonstrated greater acceptance of remote modalities, particularly telephone and e-mail communication, while neuropsychology showed a more balanced distribution across available modalities. These findings suggest that caregiver preferences may be influenced not only by the telemedicine modality itself but also by the nature and clinical requirements of the healthcare service provided.

### 3.5 Exploratory subgroup analyses

To further explore factors potentially associated with telemedicine perceptions, subgroup analyses were performed according to prior telemedicine use (Figure 3), patient sex, age group (pediatric vs. adult), and diagnosis (Dravet syndrome vs. other DEEs) (Supplementary Figures S3–S5). Overall, perceptions were largely consistent across demographic and clinical subgroups, with no clear or systematic differences observed according to patient sex, age group, or diagnosis.

**Figure 3:**
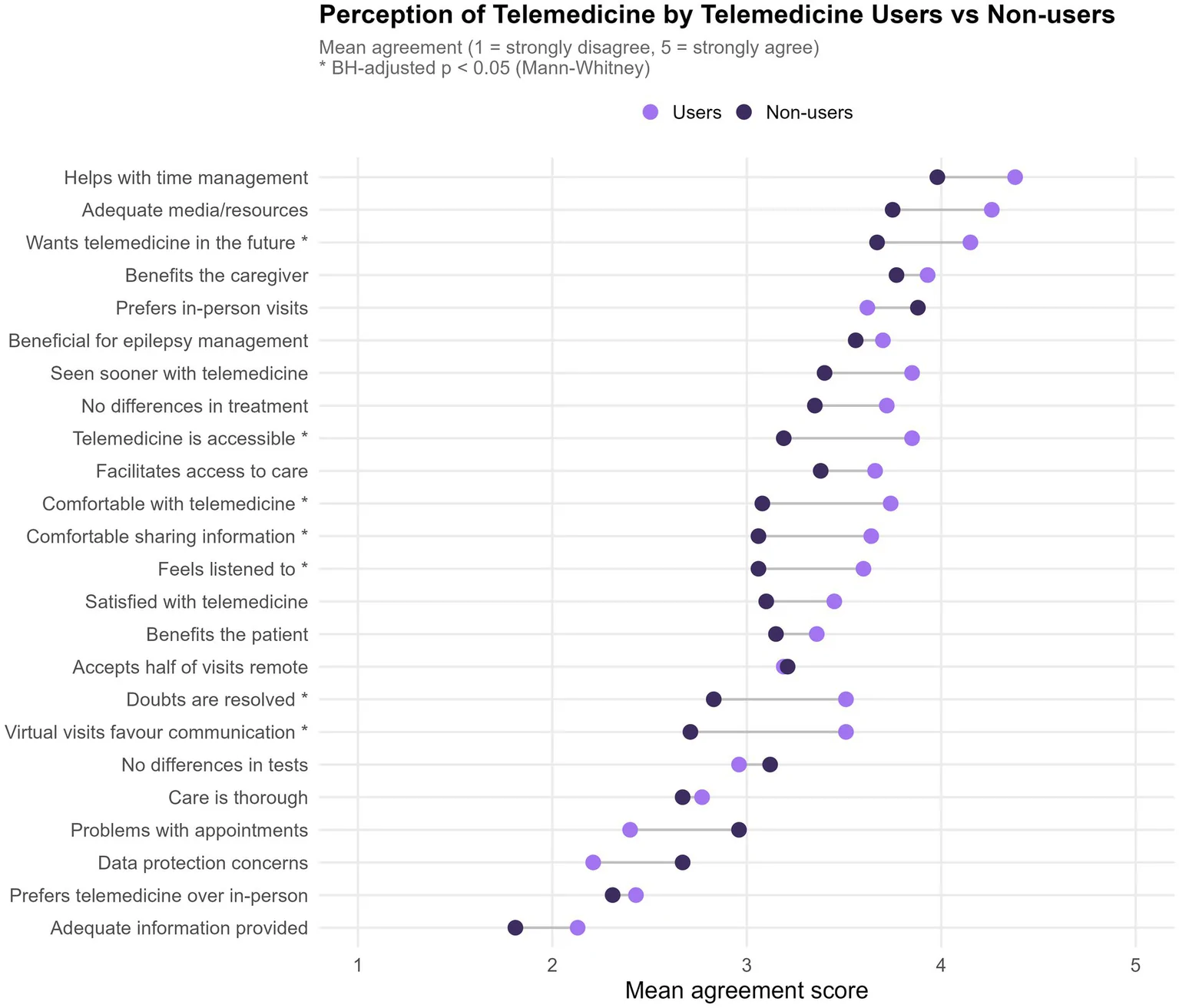
Caregiver perceptions of telemedicine according to prior telemedicine use. Mean agreement scores for telemedicine perception items among caregivers who had previously used telemedicine and those without prior telemedicine experience. Scores ranged from 1 (strongly disagree) to 5 (strongly agree). Points represent subgroup means and horizontal segments connect corresponding values between groups. Items are ordered according to overall mean agreement score. Asterisks (*) indicate statistically significant differences after Benjamini–Hochberg correction (adjusted *p* < 0.05; Mann–Whitney U test).

The most pronounced differences were observed when comparing caregivers with prior telemedicine experience to those who had never used telemedicine (Figure 3). Caregivers who had previously used telemedicine reported more favorable perceptions across several domains, including willingness to use telemedicine in the future, perceived accessibility, comfort during remote consultations, communication with healthcare professionals, and confidence that their questions and concerns could be adequately addressed during telemedicine visits. Several of these differences remained statistically significant after Benjamini–Hochberg correction.

In contrast, comparisons according to patient sex, age group, and diagnosis revealed only minor variations in individual item scores. Caregivers of patients with Dravet syndrome and those caring for individuals with other DEE subtypes reported broadly similar views regarding the perceived benefits, limitations, and future role of telemedicine. Likewise, perceptions were largely comparable between caregivers of pediatric and adult patients and between caregivers of male and female patients. These findings suggest that prior exposure to telemedicine may have a greater influence on caregiver perceptions than demographic or clinical characteristics within this cohort.

Caregiver educational level was also explored as a potential determinant of telemedicine perceptions (Supplementary Figure S6). No significant associations were observed between educational attainment and telemedicine perception, overall satisfaction, service satisfaction, or the single-item measure of satisfaction with telemedicine. Likewise, telemedicine use did not differ significantly according to educational level. Although caregivers with university education reported numerically higher scores regarding the adequacy of information received, this difference did not remain statistically significant after correction for multiple comparisons. Overall, these findings suggest that educational level was not a major determinant of telemedicine acceptance or satisfaction within this cohort.

Given the exploratory nature of these analyses and the limited sample size of some subgroups, these findings should be interpreted cautiously and considered hypothesis-generating.

### 3.6 Telemedicine in clinical trials

Thirty-nine caregivers (41%) had previously participated in a clinical trial. Among participants with experience or awareness of remote visits in clinical trials, the most highly rated advantages were comfort/convenience (rated high/very high by 72%), increased accessibility (65%), shorter waiting times and greater flexibility (both ~60%), and reduced infection risk (58%) (Figure 4A). The principal concerns were limitation of physical examination (cited by 68% of respondents), reduced personal interaction (55%), and lower supervision (42%) (Figure 4B).

**Figure 4:**
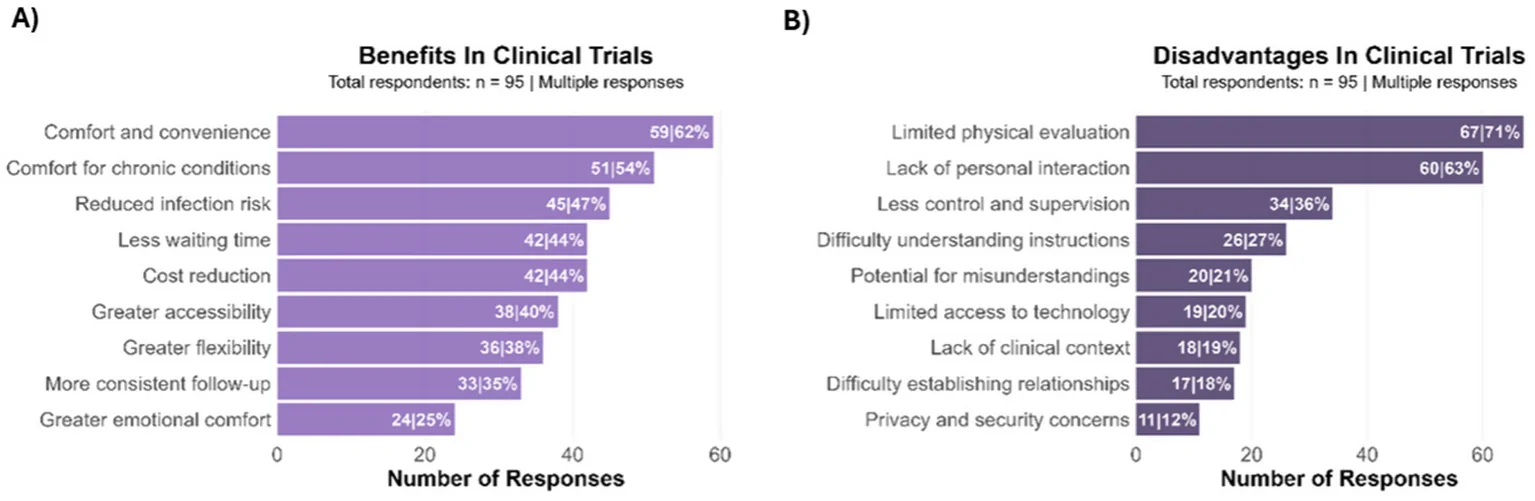
Perceptions of telemedicine in clinical trial contexts among caregivers of patients with DEEs (*n* = 95; multiple responses allowed). **(A)** Participation rates and perceived benefits of telemedicine in clinical trials. **(B)** Perceived disadvantages and barriers to telemedicine adoption in research settings.

## 4 Discussion

This study provides an overview of the sociodemographic characteristics, healthcare access patterns, and perceptions of telemedicine among caregivers of individuals with DEEs. Those caregivers reported significant unmet healthcare needs, with desired visit frequency exceeding current provision. Half had used telemedicine, mainly via telephone, but overall satisfaction was markedly lower than for in-person care, regardless of modality. Perceived benefits of telemedicine were largely caregiver-oriented (time savings, reduced travel), while clinical benefits for patients were rated substantially lower, and in-person care remained strongly preferred for most services, especially speech and occupational therapy. In clinical trial contexts, remote visits were valued for convenience, accessibility, and reduced infection risk, yet concerns persisted regarding limited physical examination, reduced personal interaction, and lower supervision. These findings underscore the tension between convenience and perceived quality in telemedicine adoption for DEEs and reveal a predominantly female caregiver population, in accordance with other Spanish studies (12, 13), with a wide age range and national geographic distribution. The limited healthcare-professional participation (*n* = 18) prevented comparative analysis and is acknowledged as a major study limitation. Nevertheless, the descriptive responses available suggest that HCPs generally viewed telemedicine positively and recognized its potential to improve access to care, while also reporting practical challenges related to connectivity, communication, and patient preparedness. These findings should be interpreted cautiously given the small sample size but provide useful contextual information complementary to caregiver perspectives.

Current ASMs used to treat patients with DEEs do not fully and effectively control seizures in the majority of patients, therefore regular outpatient consultations are important for optimizing antiseizure polytherapy, managing neuropsychiatric comorbidities, conveying psychological support and counseling on family planning (1, 14, 15). Caregivers surveyed expressed a clear desire for increased contact with HCPs, suggesting that current service provision may not fully meet their needs. Telemedicine proved to be useful in supporting remote healthcare in persons with epilepsy during the COVID-19 pandemic (16) and, according to literature previous to the pandemic, telemedicine is not inferior to in-person visits regarding seizure control, hospitalizations, emergency room visits, or medication adherence (17, 18). In our sample, the bimodal age distribution of patients, reflecting both young, recently diagnosed children and older adolescents/adults with DEEs, may partly explain the strong preference for face-to-face visits, as studies suggest that caregivers of younger children rely more heavily on in-person evaluations due to developmental and behavioral complexity. Caregivers surveyed preferred face-to-face care visits compared to telemedicine, with significantly lower satisfaction scores (*p* < 0.001), presenting skepticism about its clinical effectiveness for patients. However, telemedicine was highly valued for its logistical advantages and for easing the caregiver’s burden.

Although telemedicine was moderately adopted (50% of families), its perceived clinical value remained lower than in-person care. Satisfaction differences should however be interpreted cautiously given the low adoption of videoconferencing as the modality that typically yields higher ratings in pediatric neurology. Notably, caregivers who used videoconferencing reported numerically higher satisfaction levels than those relying exclusively on telephone-based interactions, although these differences did not reach statistical significance. This observation further supports cautious interpretation of conclusions regarding the perceived clinical value of telemedicine. Even though the use of smartphones applications can facilitate the communication and the sharing of information between caregivers and HCPs (19, 20), no telemedicine modality stood out as significantly more satisfactory, indicating that the format itself may not be the primary barrier, but rather the nature of remote interactions. Importantly, the effectiveness of telemedicine, especially newer modalities such as smartphone apps, depends heavily on the caregiver’s digital literacy and the availability of suitable electronic devices to support platforms. In contrast, educational attainment was not associated with telemedicine perception or satisfaction in our cohort. Although the study was not powered to exclude small effects, the absence of consistent differences across educational groups suggests that caregiver acceptance of telemedicine may be relatively independent of formal educational level within this population. While telemedicine has improved geographical access to care, it has not equally addressed social disparities, remaining more accessible to patients from higher socioeconomic backgrounds (5, 21, 22). Preferences for care modalities varied notably across healthcare services. Developmental and rehabilitative therapies exhibited strong resistance to digital formats, whereas consultative services, such as physiotherapy and early intervention, showed greater openness to hybrid approaches. This service-specific pattern was one of the clearest findings of the survey, carefully balancing clinical efficacy with caregiver convenience and expectations. Importantly, caregiver preferences appeared to vary according to the type of healthcare service. While neurology consultations showed greater openness to remote modalities, speech therapy and occupational therapy were more strongly associated with preferences for in-person care. This pattern suggests that acceptance of telemedicine may depend less on the communication technology used and more on whether the service requires direct observation, physical interaction, or hands-on therapeutic activities. Therefore, telemedicine implementation in DEEs should be tailored to the specific objectives and requirements of each healthcare discipline rather than adopting a uniform approach across services. Consistent with recent global evidence, hybrid models integrating telemedicine with in-person care are increasingly recommended to balance accessibility with clinical thoroughness (23).

Perceptions of telemedicine in clinical trials were included because remote or hybrid trial designs are increasingly common in rare epilepsies, and caregiver experience in these settings may influence attitudes toward telemedicine in clinical practice. Telemedicine was perceived as a facilitator of participation, offering increased comfort, improved accessibility, and reduced logistical barriers. However, notable concerns were raised regarding the absence of physical examinations, limited personal interaction, and reduced supervision, factors that may undermine trust and engagement in remote research settings. These findings align with the broader understanding that, although randomized controlled trials remain the gold standard for data quality, standardized protocols, and patient safety, they are often costly, time-consuming, and geographically restrictive (24). This is particularly problematic in the context of rare diseases such as DEEs, where patient populations are dispersed and access to specialized centers is limited. Ongoing clinical trials are currently exploring whether telemedicine can enhance the quality and efficiency of research processes. These efforts may pave the way for integrating telemedicine into clinical trial settings, potentially expanding participation and improving accessibility, particularly for patients with rare and geographically dispersed conditions (25, 26).

Several limitations should be acknowledged. First, the study relied on a convenience sample recruited primarily through patient advocacy organizations, which may have introduced selection bias by preferentially reaching families who are more engaged, connected to support networks, and potentially more interested in telemedicine-related topics. Although participants were recruited from 28 Spanish provinces through nationally disseminated recruitment channels, the sample should not be considered representative of the overall Spanish DEE population. Accordingly, the term “nationwide” refers to the geographical reach of recruitment and participation rather than population representativeness. In addition, Dravet syndrome accounted for the majority of responses, which may limit the generalizability of the findings to other DEE subtypes with different clinical characteristics and care needs. The cross-sectional design prevents assessment of changes in telemedicine perceptions over time, and no formal sample size or power calculation was performed, as this was conceived as an exploratory survey study; therefore, findings should be considered descriptive and hypothesis-generating. Although educational level was explored, the subgroup with lower educational attainment was small. Furthermore, information regarding socioeconomic status, household income, digital literacy, and distance to specialized healthcare facilities was not collected, preventing assessment of their potential influence on telemedicine perceptions. The survey instrument was purpose-designed for this study and underwent expert review and cognitive pretesting, but it did not undergo formal psychometric validation. Consequently, the reliability and construct validity of some measures remain uncertain and findings should be interpreted accordingly. The low number of HCPs who completed the survey (*n* = 18) precluded meaningful comparisons between caregiver and professional perspectives. Interpretation is further limited by the low adoption of videoconferencing, with most reported telemedicine experiences relying on telephone calls or e-mail communication, thus restricting the extent to which these findings can be extrapolated to comprehensive audiovisual telemedicine models. Finally, the study was conducted exclusively in Spain and within a specific healthcare context, which may limit transferability of the results to other countries and healthcare systems.

To synthesize the findings of this study, a conceptual framework was developed as an exploratory synthesis of descriptive patterns observed in the survey, rather than as an inferential model (Figure 5). It should therefore be interpreted as a preliminary representation of caregiver-reported domains. This framework should not be interpreted as a validated model, but rather as a visual synthesis of themes emerging from the present survey. It summarizes the main domains affected by telemedicine in the care of patients with DEEs, as well as perceived benefits, barriers, and recommendations for future implementation. This framework integrates caregiver perspectives, service-specific considerations, and implications for clinical trials, providing a visual representation of the complex factors influencing telemedicine adoption.

**Figure 5:**
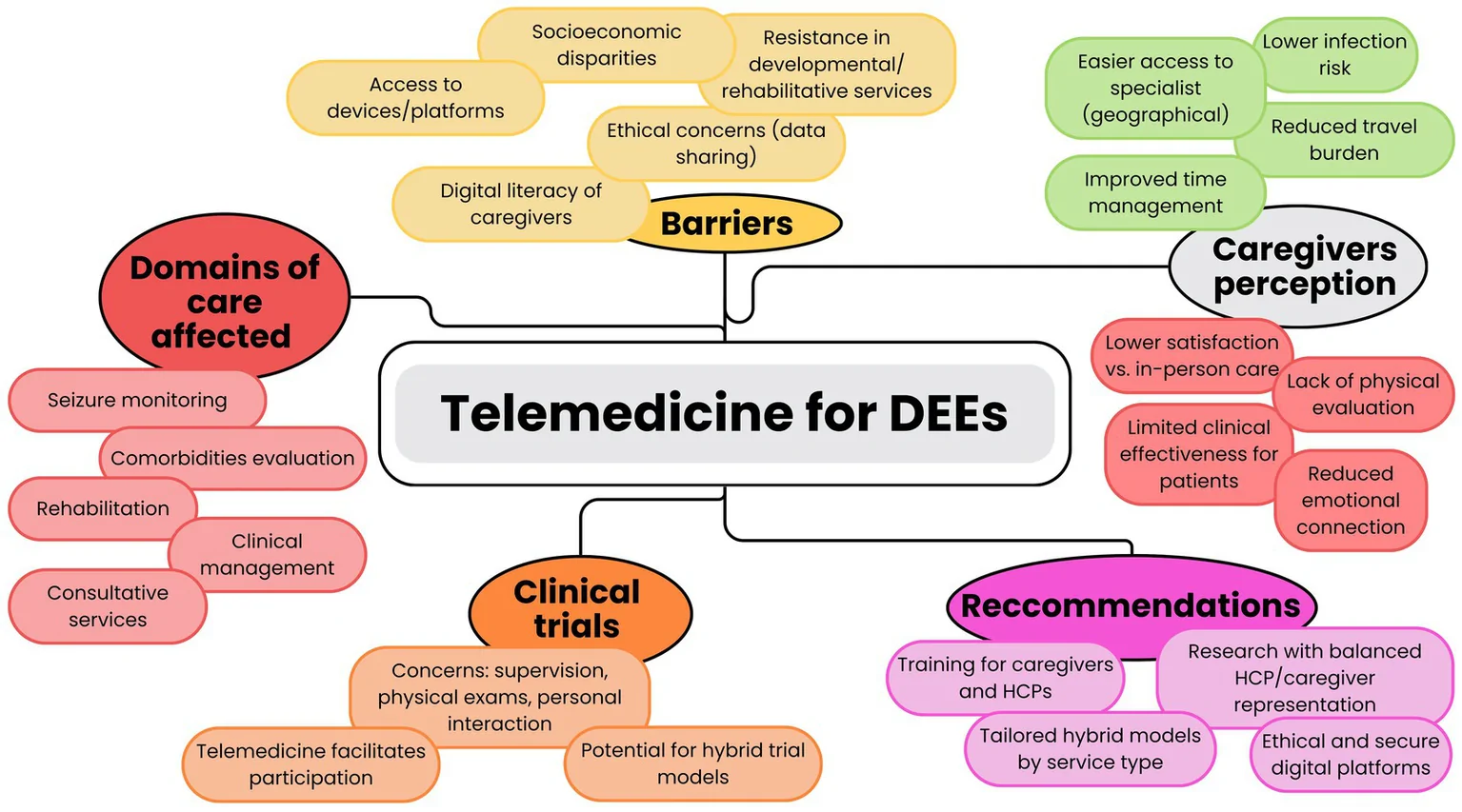
Conceptual framework of telemedicine in DEEs. The diagram illustrates key domains of care impacted by telemedicine, caregiver perceptions (advantages and limitations), barriers to adoption, implications for clinical trial participation, and recommendations for future strategies, including tailored hybrid models and training initiatives. The framework is intended as an exploratory visual synthesis of themes emerging from the survey and should not be interpreted as a validated conceptual or predictive model.

## 5 Conclusion

The findings of this nationwide survey highlight that caregivers of individuals with DEEs value the logistical advantages of telemedicine, particularly in reducing travel demands, time burden, and perceived infection risk. However, surveyed caregivers generally perceived in-person care as providing greater clinical value and being better suited to address the complex healthcare needs associated with DEEs. These findings should be interpreted cautiously, as telemedicine in our sample consisted predominantly of telephone calls and e-mail communication, with limited use of videoconferencing, which is generally considered the reference modality for remote neurological care. Furthermore, the exploratory nature of the study, the absence of a formal sample-size calculation, and the use of a convenience sample limit the strength and generalizability of the conclusions. These factors also restrict the extent to which caregiver perceptions observed in this study can be extrapolated to more comprehensive telemedicine models, particularly for complex DEE-related care requiring physical examination, behavioral observation, and multidisciplinary assessment.

Overall, within this cohort, caregivers tended to view telemedicine as a complementary component of care rather than a complete alternative to face-to-face services. The findings support a tailored approach in which remote and in-person modalities are combined according to the characteristics of each healthcare service and the preferences of families. In-person care may remain particularly important for high-complexity assessments and rehabilitative interventions, while telemedicine may offer value for follow-up, coordination, administrative processes, and supportive care. Future research should focus on evaluating structured hybrid care models and expanding access to high-quality telemedicine tools, particularly videoconferencing, to better understand their potential role in the management of individuals with DEEs.

## Data Availability

The raw data supporting the conclusions of this article will be made available by the authors, without undue reservation.
