# Supplementary material for: Caregiver perceptions of telemedicine in developmental and epileptic encephalopathies: a nationwide Spanish survey
Source: Front Neurol. 2026 Sep 28;17:1842836. doi: 10.3389/fneur.2026.1842836 (PMC13654694; doi:10.3389/fneur.2026.1842836)
Supplement: Supplementary file 1 [file Table_1.DOCX]

Supplementary Material

# 1 Supplementary Figures and Tables

## 1.1 Supplementary Figures

**Supplementary Figure 1. Descriptive findings from healthcare professionals regarding telemedicine in DEEs. (A)** Healthcare professionals' perceptions of telemedicine. Distribution of responses on a 5-point Likert scale ranging from strongly disagree (1) to strongly agree (5) among healthcare professionals who completed the survey (n = 15 with complete response data). Items evaluate attitudes toward telemedicine, perceived benefits, accessibility, workload, satisfaction, and future use of remote consultations for patients with DEEs. **(B)** Difficulties experienced during remote consultations. Horizontal bar chart showing the proportion and number of healthcare professionals reporting technical, logistical, or communication-related challenges during telemedicine visits (n = 14 professionals performing remote consultations). Multiple responses were allowed; therefore, percentages do not total 100%.

**Supplementary Figure 2. Preferred healthcare modalities by service type.** Horizontal stacked bar chart illustrating the preferred healthcare delivery modalities (in-person, phone, email, video) reported by participants for each of four services: Neurology, Neuropsychology, Occupational Therapy, and Speech Therapy. In-person visits were the most desired modality across all services, particularly in Neurology. Speech and Occupational Therapy showed more interest in remote formats (phone or email) compared to Neurology. Neuropsychology presented the most even distribution among all modalities.

**Supplementary Figure 3. Caregiver perceptions of telemedicine according to patient sex.** Mean agreement scores for telemedicine perception items among caregivers of male and female patients with DEEs. Scores ranged from 1 (strongly disagree) to 5 (strongly agree). Points represent subgroup means and horizontal segments connect corresponding values between groups. Items are ordered according to overall mean agreement score. Asterisks (*) indicate statistically significant differences after Benjamini–Hochberg correction (adjusted p < 0.05; Mann–Whitney U test).

**Supplementary Figure 4. Caregiver perceptions of telemedicine according to patient age group.** Mean agreement scores for telemedicine perception items among caregivers of pediatric (<18 years) and adult (≥18 years) patients with DEEs. Scores ranged from 1 (strongly disagree) to 5 (strongly agree). Points represent subgroup means and horizontal segments connect corresponding values between groups. Items are ordered according to overall mean agreement score. Asterisks (*) indicate statistically significant differences after Benjamini–Hochberg correction (adjusted p < 0.05; Mann–Whitney U test).

**Supplementary Figure 5. Caregiver perceptions of telemedicine according to diagnosis group.** Mean agreement scores for telemedicine perception items among caregivers of individuals with Dravet syndrome and other genetic DEE subtypes. Scores ranged from 1 (strongly disagree) to 5 (strongly agree). Points represent subgroup means and horizontal segments connect corresponding values between groups. Items are ordered according to overall mean agreement score. Asterisks (*) indicate statistically significant differences after Benjamini–Hochberg correction (adjusted p < 0.05; Mann–Whitney U test).

**Supplementary Figure 6. Telemedicine perception and satisfaction by caregiver educational level** (n= 95). **(A)** Distribution of the three composite indices and of the single satisfaction item across the three educational strata; boxes show the median and interquartile range and points are individual caregivers. **(B)** Item-level comparison between university and non-university caregivers for the 22 perception items, expressed as rank-biserial correlations with 95% bootstrap confidence intervals; positive values indicate higher agreement among university-educated caregivers and the dashed line marks the null. No comparison remained statistically significant after Holm correction, and all confidence intervals include zero, indicating that perception of and satisfaction with telemedicine were homogeneous across educational levels in this cohort.

## 1.2 Supplementary Tables

**Supplementary Table 1. Baseline demographic and clinical characteristics of patients with DEEs (n = 95).**

| **Category** | **Variable** | **Value** |
| --- | --- | --- |
| **Sociodemographics** | Patient age | Mean 14.01 years (SD 11.11), median 11, range 0–54 (N=95) |
|  | Caregiver age | Mean 47.13 (SD 9.54), range 27–73 (N=95) |
|  | Sex | Male 49 (51.6%), Female 46 (48.4%) |
|  | Geographic distribution | Respondents from 28 provinces; most frequent: Madrid (20%), Barcelona (10.5%) |
| **Clinical characteristics** | Diagnosis | Dravet 71.6%; KCNQ2 11.6%; unspecified DEE 5.3%; Cdkl5 2.1%; Rett 2.1%; West 2.1%; others ≤1% |
|  | Disability grade | Grade V 53.7%; Grade IV 22.1%; Grade III 14.7%; Grade II 1.1%; Unknown 8.4% |
|  | Dependence grade | High 62.1%; Severe 15.8%; Moderate 8.4%; Unknown 13.7% |
|  | Age at diagnosis | Mean 3.32 years (SD 5.46), median 1, range 0–33 (N=95) |
|  | Age at first manifestations | Mean 4.01 years (SD 2.93), median 4, range 0–12 (N=87) |
|  | Age at first seizures | Mean 5.28 years (SD 6.86), median 4, range 0–60 (N=87) |
|  | Seizure types | Tonic‑clonic 38.39%; Focal 17.06%; Absence 14.69%; Myoclonic 13.27%; Tonic 9.48%; Atonic 4.27%; Other 2.84% |
|  | Number of seizure types | 1 type: 46.32%; 2 types: 21.05%; 3 types: 14.74%; ≥4 types: 17.9% |
|  | Annual seizure frequency | Median 12 (IQR 3–48); mean 48.96 (SD 87.87), range 1–365 (N=85) |
|  | Comorbidities | Speech/language/cognitive 28.01%; Motor/orthopedic 22.48%; Behavioral/ASD 18.89%; Sleep 14.33%; Digestive 8.79%; Resp/cardiac 4.56%; Other 2.61%; None 0.33% |
| **Pharmacological treatment** | Use of rescue medication | Yes 31.58%; No 68.42% |
|  | Number of medications | 1:1.05%;2:22.11%;3:3.16%;4:24.21%;5:12.63%;6:24.21%;8:6.32%;9:2.11% |
|  | Treatment categories | ASMs 40.61%; Rescue 22.71%; Sleep 14.85%; Digestive 8.73%; Neuroleptics 3.49%; Others 9.61% |
|  | Common combinations | ASM-only 22.11%; ASM+rescue 15.79%; ASM+rescue+sleep 14.74% |
| **Non-pharmacological therapy** | Any therapy used | Yes 93.68%; No 6.32% |
|  | Most common therapies | Speech 19.25%; Physio 18.63%; Orthopedics 11.49%; OT 9.63%; Early intervention 7.45%; Aquatic 6.52%; Nutrition 5.59%; Psych 4.66%; Neuropsych 4.35%; Equine 4.35%; Optometry 3.42% |
|  | Number of therapies | 2:20%;3:20%;4:14.74%;5:12.63%;6:9.47%;None:6.32% |
| **Hospital use** | Hospitalizations (epilepsy-related) | Significantly higher in infants (90.91%) |
|  | Hospitalizations (non-epilepsy) | Highest in infants (54.55%) |
|  | Medication collection | Hospital 73.17%; Not prescribed 25.61%; Home delivery 1.22% |
|  | Transportation | Family vehicle 63%; Walking 10%; Home-visit 10%; Varies 9%; Public 6.7%; Taxi <1% |
|  | Accompanying person | Parent 84.26%; Grandparents 5.56%; Paid 4.63%; Siblings 1.85%; Other 3.7% |

*Abbreviations: DEE, developmental and epileptic encephalopathy; HCP, healthcare professional; SD, standard deviation; IQR, interquartile range.

# 2 Questionnaires

This supplementary contains the complete versions of the survey instruments administered to healthcare professionals (2.1) and caregivers (2.2), including all items, response formats and distribution wording.

## 2.1 Survey for professionals

1. How many people diagnosed with DEE do you see per month?

- None (please do not complete the survey). More information on EDE and Dravet syndrome is available at dravetfoundation.eu
- Less than one per month, but at least two per year
- Weekly: dropdown menu (1–100)
- Monthly: dropdown menu (1–100)
- Yearly: dropdown menu (1–100)

2. Province of practice: (dropdown list)
3. Age:

- 25–34
- 35–44
- 45–55
- 55

4. Gender:

- Female
- Male
- Non-binary
- Prefer not to answer

5. Years of experience treating DEE patients: (dropdown 0–50)
6. Number of DEE patients seen per month: (dropdown 0–100)
7. Type of healthcare facility:

- Public hospital
- Private hospital
- Health center
- Private clinic
- Contracted center
- Primary care
- Medical office
- Rehabilitation center
- Dental clinic
- Disability care center

8. Population served:

- Adults
- Children

9. Epilepsy consultation type:

- Dedicated
- Non-dedicated

10. Do you conduct remote visits with DEE patients?

- Yes
- No

11. If yes, type of remote visit:

- Telephone
- Virtual/teleconsultation

12. Of your total monthly DEE visits, how many are:

- In-person (dropdown 0–100)
- Telephone (dropdown 0–100)
- Virtual (dropdown 0–100)

13. Administrative support for scheduling visits:

- With support
- Self-organized / no support

14. Have you received specific training for remote visits?

- Yes
- No

15. If no, would you like to receive such training?

- Yes
- No

16. Rate technical difficulties during remote visits (1 = Never, 10 = Always):

- Scale 1–10

17. Main difficulties encountered:

- Connectivity issues
- Patient/family unprepared
- Multiple call attempts needed
- Patient/family did not understand instructions
- All of the above
- No difficulties

18. Have you made medication changes during remote visits?

- Yes
- No

19. Rate change in duration of remote vs. in-person visits (1 = Shorter, 10 = Longer):

- Scale 1–10

20. Attitudes Toward Telemedicine: Rate agreement (4 = Strongly agree, 0 = Strongly disagree):

- I always prefer in-person visits
- I prefer telemedicine over in-person visits
- I would be satisfied conducting half of my visits remotely
- My workload has increased since using telemedicine
- I feel the patient/family listens and understands during remote visits
- I feel comfortable during remote visits
- Telemedicine improves access to healthcare services
- Telemedicine allows earlier patient contact
- The time allocated for remote visits is sufficient
- I am concerned about data privacy during remote visits
- Overall, I am satisfied with telemedicine
- I am open to remote visits in the future
- I believe teleneurology visits are beneficial for epilepsy patients

21. Diagnostic and Consultation Follow-Up: Rate reception of tests and referrals requested during remote visits (1 = Same as in-person, 10 = Delayed):

- Scale 1–10

22. Can you resolve patient/family doubts during remote visits? (1 = Never, 10 = Always):

- Scale 1–10

If score is 0–5, select reason(s):

- Technical limitations
- Nature of the medical issue
- Need for physical evaluation
- Lack of personal interaction
- Privacy concerns
- Communication barriers
- Medical urgency
- Patient preference
- Legal or ethical concerns
- Lack of continuity
- Other

23. Do you believe telemedicine benefits DEE patients? (1 = No benefit, 10 = Full benefit):

- Scale 1–10

If score is 5–10, select reason(s):

- Improved access
- Regular follow-up
- Closer monitoring during acute episodes
- Reduced infection risk
- Patient convenience
- Remote education
- Geographic reach
- Improved adherence
- Cost reduction
- Other

If score is 0–5, select reason(s):

- Lack of personal interaction
- Technical limitations
- Medical complexity
- Privacy concerns
- Communication barriers
- Patient preference
- Other

24. Do you believe healthcare professionals can provide quality care via telemedicine?

- Yes
- No
- Not sure

25. Clinical Trials: Are clinical trials conducted at your center?

- Yes
- No

26. Most trials at your center are:

- In-person
- Remote
- Hybrid

27. Benefits of remote clinical trial care (ranked):

- Convenience
- Accessibility
- Cost reduction
- Flexibility
- Consistent follow-up
- Shorter wait times
- Comfort for chronic conditions
- Reduced infection risk
- Emotional comfort

28. Drawbacks of remote clinical trial care (ranked):

- Lack of personal interaction
- Limited physical evaluation
- Less supervision
- Limited tech access
- Lack of clinical context
- Privacy/security concerns
- Difficulty forming relationships
- Instruction comprehension issues
- Lack of emotional support
- Potential misunderstandings

29. Benefits of hybrid clinical trial care (ranked):

- Accessibility
- Lower costs
- Continuity of care
- Patient comfort
- Reduced infection exposure
- Flexibility
- Improved communication
- Proactive health management

30. Drawbacks of hybrid clinical trial care (ranked):

- Technical difficulties
- Privacy concerns
- Limited physical exams
- Diagnostic errors
- Depersonalization
- Inequity in access
- Overreliance on technology

31. Are data collected during remote trial visits rigorous?

- Yes
- As rigorous as in-person
- More rigorous than in-person
- No
- Less rigorous than in-person
- Please explain (open text)

32. Does telemedicine improve recruitment in clinical trials?

- Yes
- No
- Not sure

33. Does telemedicine improve patient retention in clinical trials?

- Yes
- No
- Not sure

34. What additional data could be collected via telemedicine that are currently not captured?

- Open text

35. What aspects are missing in current telemedicine practices?

- Open text

## 2.2 Survey for caregivers

1. Are you of legal age, a relative or caregiver of a person diagnosed with DEE and residing in Spain?

- Yes, please procDEE with the questionnaire
- No, please do not complete the questionnaire. You can find more information about DEE at https://www.dravetfoundation.eu/

2. In which province do you reside? (Dropdown list)
3. What is your relationship with the person diagnosed with DEE?

- Parent/Legal guardian
- Sibling
- Grandparent
- Caregiver
- Other

4. What is your gender?

- Male
- Female
- Non-binary
- Prefer not to say

5. What is your age (in years)? (Dropdown menu from 0 to 100)
6. Education level:

- No formal education
- Primary education
- Secondary education
- High school
- Vocational training
- University education

7. Employment status

- Employed
- Unemployed
- Retired

8. Work schedule (if employed)

- Full-time (40–37.5 hours/week)
- Reduced schedule (30 hours/week)
- Part-time (20–25 hours/week)

9. Are you the primary caregiver?

- Yes
- No

10. What is the gender identity of the person with DEE?

- Male
- Female
- Non-binary
- Prefer not to say

11. What is the age of the person with DEE (in years)? (Dropdown menu from 0 to 100)
12. Level of dependency of the person with DEE: On a scale from 1 to 10 where 1 = severe dependency (requires help for most daily activities) and 10 = independent/autonomous
13. At what age was the person diagnosed with DEE? (Dropdown of years and months)
14. Diagnosis of the patient (dropdown menu based on Wirrell et al. 2011)
15. Indicate the age (in years and months) of onset of the first clinical manifestations of the person with DEE.
16. Indicate the age (in years and months) of onset of the first seizures of the person with DEE. (Removable from integers 0 to 10 years and from 1 to 11 months)
17. Indicate the types of seizures that the person with DEE currently presents (Drop-down with types of seizures: tonic-clonic, partial or focal, tonic, myoclonic, absences, atonic)
18. If the patient has daily seizures, indicate the number of seizures per day. (Fold-down of whole numbers from 1 to 10)
19. If weekly seizures, indicate the number of seizures per day. (Fold-down of whole numbers from 1 to 10)
20. If monthly seizures, indicate the number of seizures per day. (Fold-down of whole numbers from 1 to 10)
21. Indicate which comorbidities associated with the DEE, if any, (they may mark more than 1):

- Digestive problems, malnutrition, etc.
- Respiratory tract infections, cardiovascular problems, etc.
- Motor problems, delayed body growth, and/or orthopedic conditions
- Speech and language delay, cognitive disturbances,
- Behavioral problems, deficiency in social relationships, characteristics of ASD, etc.
- Sleep disturbances
- Other

22. Has the person been hospitalized for more than 24 hours due to prolonged seizures in the last 6 months?

- Yes
- No

23. Has the person been hospitalized for reasons other than seizures related to DEE in the last 6 months?

- Yes
- No

24. Has the person experienced recurrent seizures or seizures lasting more than one hour?

- Yes
- No

25. Does the person usually require rescue medication during seizures (e.g., buccolam, noiafren or other benzodiazepines)?

- Yes
- No

26. What pharmacological treatments does the person with DEE follow?

- ASMs: valproic, clobazam, clonazepam, topiramate, stiripentol, zonisamide, levetiracetam, perampanel, cannabidiol, fenfluramine, others.
- Rescue medication: midazolam, diazepam, clonazepam, valproic, phenobarbital, others.
- Neuroleptics (for behavior): risperidone, chlorpromazine, others.
- For insomnia: melatonin, tranxilium, lorazepam, others.
- Digestive system: laxatives, omeprazole, others.
- Other drugs

27. Does the person with DEE require complementary treatments (e.g., speech therapy, physiotherapy, orthopedics, dentistry) in addition to medication?

- Yes
- No

28. If so, what clinical treatment is involved? (you can check more than one)

- Early care
- Audiological adaptations
- Auditory stimulation
- Physiotherapy or psychomotor therapy
- Speech therapy
- Neuropsychological intervention
- Optics and optometry
- Orthopaedics
- Clinical Psychology
- Dietetic-nutritional monitoring
- Aquatic Therapy/Hydrotherapy
- Occupational therapy
- Equine therapy or therapy with horses
- Other

29. Do you notice fluid communication between the different specialists who treat your child? Answer on a scale of 1 to 10 where 1 is "very difficult communication: communication with obstacles, lack of clarity." and 10 is "very fluent communication: exceptional fluency and clarity in communication."
30. Have you ever tried to group appointments on the same day to avoid multiple trips to the hospital?

- Yes
- No

31. If so, how have you perceived the treatment you received? On a scale of 1 to 10 where 1 is "I have not perceived support or understanding." and 10 is "I have felt support and understanding in my difficulties."
32. How do you think coordination between different levels of care and specialists treating your child could be improved to reduce the impact on your child’s and family’s quality of life?

- Improve communication between professionals
- Improve communication at administrative levels
- Liaison posts between levels of care
- Interdisciplinary working groups
- Facilitate the flexibility of professionals to do telemedicine

33. How is the person with DEE receiving care? (for each choice drop-down menu with video call/phone/email/face-to-face/not applicable)

- Primary care, (modality)
- Neurology Specialist, (modality)
- Audiological adaptations, (modality)
- Auditory stimulation, (modality)
- Physiotherapy, (modality)
- Speech therapy intervention, (modality)
- Neuropsychological intervention, (modality)
- Optics and optometry, (modality)
- Orthopedics, (modality)
- Clinical psychology, (modality)
- Dietetic-nutritional follow-up, (modality)
- Aquatic therapy/hydrotherapy, (modality)
- Occupational therapy, (modality)
- Speech therapy (modality)
- Other, (modality)

34. Would you like to see the care model changed in any of them? video/phone/email/face-to-face/ not applicable)

- Primary care, (modality)
- Neurology Specialist, (modality)
- Audiological adaptations (modality)
- Auditory stimulation, (modality)
- Physiotherapy, (modality)
- Speech therapy intervention, (modality)
- Neuropsychological intervention, (modality)
- Optics and optometry, (modality)
- Orthopedics, (modality)
- Clinical psychology, (modality)
- Dietetic-nutritional follow-up, (modality)
- Aquatic therapy/hydrotherapy, (modality)
- Occupational therapy, (modality)
- Other, (modality)

35. On average, how many different times per week does the person with DEE attend all therapies?
36. If you could choose, how many times per week would you like the person with DEE to attend all therapies?
37. How does the person with DEE travel to therapies?

- On foot
- Public transport
- Taxi
- Family Vehicle
- Therapists come home
- It depends on the therapy

38. Does someone accompany the person with DEE to therapy sessions?

- Yes
- No

39. Do you think that travel to health care facilities is a benefit or difficulty for the person with DEE? Answer on a scale of 1 to 10 where 1 is "difficulty", 5 is "no change" and 10 is "benefit".
40. Has the person with DEE received telemedicine care?

- Yes
- No

41. How do you usually access telemedicine?

- Mobile App
- WhatsApp
- Email
- Video conferencing (Skype/Teams/others)
- Mobile Call
- Other

42. In the following questions, answer the corresponding option depending on whether you strongly agree (4), partially agree (3), neither agree nor disagree (2), partially disagree (1) or strongly disagree (0).

- I always prefer face-to-face visits
- I prefer telemedicine to in-person visit
- I consider telemedicine to be accessible to me
- I have received information about the use of telemedicine
- I have the means to make use of telemedicine
- During telemedicine visits, the patient is approached thoroughly
- The use of telemedicine benefits the patient with DEE
- The use of telemedicine benefits the parent/caregiver of the person with DEE
- I would be happy to make half of the visits in a non-face-to-face manner.
- Virtual visits have favored communication with the neurologist
- I solve all my doubts in telemedicine visits
- I feel that the neurologist listens and understands me during the televisit
- I feel comfortable in the televisit
- Telemedicine facilitates access to Health Services
- I have not had significant differences in treatment with respect to face-to-face appointments (change of drugs, duration, frequency of visits)
- I have not had differences in the performance of tests on the person with DEE (video/electroencephalograms, neuroimaging, etc.)
- I believe that the use of telemedicine allows you to see the neurologist sooner
- During telemedicine visits, I am concerned about data protection and privacy (that others may hear or see us)
- During telemedicine visits, I feel comfortable sharing videos or photos of the patient
- Overall, I am satisfied with the use of telemedicine
- I am open to telematic visits in the future
- I think teleneurology visits to patients with epilepsy are beneficial
- Telemedicine helps us to manage time, reducing travel times, waiting, etc.
- I've had issues with telemedicine appointments (connectivity, waiting, lack of understanding, etc.)

43. Degree of satisfaction with the organisation of telematic consultations. You can answer: Very satisfied (6), quite satisfied (5), satisfied (4), somewhat dissatisfied (3), dissatisfied (2), very dissatisfied (1) or not applicable (N/A)
44. How satisfied are you with the following aspects of telemedicine visits?

- Time to answer all your questions
- Consideration of your individual needs
- Inclusion of your ideas in the treatment process.
- Understanding and empathy from staff.

45. How satisfied are you with the organization of the epilepsy clinic in the context of telemedicine visits?

- Appointment allocation
- Appointment Schedule Compliance
- Willingness to help and friendliness of staff
- Continuity of contact personnel
- Atmosphere during the consultation
- Planning and arranging visits and tests

46. As for hospital-dispensed medicines, how do you get them? (you can choose more than one)

- I go to the hospital to pick them up
- I receive them at home
- I receive them elsewhere pharmacy/health center
- We have not been prescribed it

47. Are you currently participating or have you participated in a clinical trial?

- Yes
- No

48. What was the format of the clinical trial?

- Face
- Telematic
- Hybrid

49. If the trial was or is telematic, through which medium?

- Video conferencing (Zoom, Teams, Skype, other)
- Call
- Email
- Other

50. While participating in the trial, has the format changed?

- Yes, from telematic to face-to-face
- Yes, from face-to-face to telematic
- Yes, hybrid
- No

51. What benefits do you think telemedicine offers in clinical trials?

- Comfort and convenience
- Increased accessibility
- Cost reduction
- Greater flexibility
- More consistent tracking
- Shorter waiting time
- Increased comfort for people with chronic conditions
- Reduced risk of contagion
- Increased emotional comfort

52. What drawbacks do you think telemedicine presents in clinical trials?

- Lack of personal interaction
- Limited physical evaluation
- Less control and supervision compared to face-to-face visits
- Limited access to technology resources
- Lack of clinical context
- Potential privacy and security issues
- Limitation in the ability to establish relationships
- Difficulty understanding instructions
- Lack of emotional support
- Potential for misunderstandings

53. How would you prefer to access telemedicine?

- Mobile App
- WhatsApp
- Email
- Video conferencing (Skype/Teams/others)
- Mobile Call
- Other
